# Maximal Oxygen Consumption Is Negatively Associated with Fat Mass in Facioscapulohumeral Dystrophy

**DOI:** 10.3390/ijerph21080979

**Published:** 2024-07-26

**Authors:** Oscar Crisafulli, Luca Grattarola, Giorgio Bottoni, Jessica Lacetera, Emanuela Lavaselli, Matteo Beretta-Piccoli, Rossella Tupler, Emiliano Soldini, Giuseppe D’Antona

**Affiliations:** 1 CRIAMS-Sport Medicine Centre Voghera, University of Pavia, 27058 Voghera, Italy; 2 Rehabilitation Research Laboratory 2rLab, Department of Business Economics, Health and Social Care, University of Applied Sciences and Arts of Southern Switzerland, 6928 Manno, Switzerland; 3 Department of Life Sciences, University of Modena and Reggio Emilia, 41125 Modena, Italy; 4 Competence Centre for Healthcare Practices and Policies, Department of Business Economics, Health and Social Care, University of Applied Sciences and Arts of Southern Switzerland, 6928 Manno, Switzerland; 5 Department of Public Health, Experimental and Forensic Medicine, University of Pavia, 27058 Voghera, Italy

**Keywords:** FSHD, body composition, muscular dystrophy, aerobic fitness, clinical stratification

## Abstract

Facioscapulohumeral dystrophy (FSHD) leads to progressive changes in body composition such as loss of muscle mass and increase in adiposity. In healthy subjects, anthropometric parameters are associated with the maximum volume of oxygen consumed per minute (VO_2_max), which is a health and function indicator in several populations of subjects, both healthy and pathological. Since VO_2_max can be difficult to test in patients with FSHD due to exercise intolerance, the identification of associated anthropometric parameters could provide new easily obtainable elements for the patients’ clinical stratification. The aim of this study was to evaluate whether anthropometric and body composition parameters are associated with VO_2_max in patients with FSHD. A total of 22 subjects with a molecular genetics-based diagnosis of FSHD (6 females, 16 males, mean age of 35.18 years) were recruited for the study. VO_2_max was measured by cardiopulmonary exercise tests (CPETs) on a cycle ergometer, utilizing a step incremental technique (15 Watts (W) every 30 s). Weight (Kg) and height (m) were obtained and utilized to calculate body mass index (BMI). Body composition parameters (fat mass (FM), fat free mass (FFM), and body cell mass (BCM)) were obtained by bioelectrical impedance analysis (BIA). Significant negative associations were found between VO_2_max and FM (Spearman correlation coefficient (SCC) −0.712), BMI (SCC −0.673), age (SCC −0.480), and weight (SCC −0.634), unlike FFM and BCM. Our results indicate that FM, BMI, age, and body weight are negatively associated with VO_2_max in patients with FSHD. This evidence may help practitioners to better stratify patients with FSHD.

## 1. Introduction

Facioscapulohumeral dystrophy (FSHD, OMIM #158900) is the third most common form of muscular dystrophy after Duchenne muscular dystrophy and myotonic dystrophy type 1 [1], with an estimated prevalence of between 1:15,000 and 1:20,000 [2]. The name of the disease is due to the fact that it manifests itself primarily with weakness of the face, shoulder, and arm muscles. The diagnosis of FSHD is determined by a combination of genetic and clinical features, as well as the exclusion of disorders that share similar characteristics. Genetically, it is typically associated with deletion of an integral number of tandem 3.3 kb units of the polymorphic D4Z4 repeat array at 4q35 [3]. Notably, D4Z4 alleles with fewer than 10 repeat units in association with the 4qA polymorphism are considered pathogenic [4]. A peculiar clinical feature of FSHD is the progressive loss of fat free mass (FFM) and the associated increase in fat mass (FM). Indeed, Vera et al. [5] reported that patients with FSHD commonly meet the definition of sarcopenic obesity, a condition that combines the key features of sarcopenia with an increased presence of adiposity. This change in body composition leads to face, upper extremity, arm, lower leg, and hip girdle muscle weakness, which often manifests asymmetrically [6]. Another pathological trait is marked fatigue, which can be induced by loss of muscle strength, physical overachieving or underachieving, and stress, negatively impacting quality of life and social participation [7,8]. Apart from rare cases that present respiratory impairment [9], life expectancy is not reduced [10]. However, the disease has a wide phenotypic spectrum, with heterogeneous patterns of symptoms and progression [11]. To overcome such heterogeneity in the clinical presentation, patients are classified according to the Complete Clinical Evaluation form (CCEF), which categorizes subjects based on: facial and scapular girdle muscle weakness (category A); muscle weakness limited to the scapular girdle or facial muscles (category B); no symptoms (category C); and myopathic phenotype presenting inconsistent clinical features with the canonical FSHD phenotype (category D) [12]. In FSHD, the association between body composition and functional outcomes has been underlined in several available studies [13,14,15]. For instance, Skalsky et al. [13] reported that patients show increased regional fat mass, decreased regional lean mass, and loss of strength. Similarly, lower limb muscle degeneration is proposed as a main cause of gait impairment [14]. Moreover, a study of Vera et al. [15] pointed out that the loss of lean mass due to the disease leads to exercise intolerance, as evidenced by a lower VO_2_peak and elevated symptoms of dyspnea and fatigue during submaximal exercise compared to healthy controls. The connection between body cell mass (BCM) (the metabolically active subcomponent of FFM) [16] and functional outcomes has not been investigated in patients with FSHD yet.

Literature data suggest that, in healthy subjects, body composition parameters are associated with the maximal oxygen uptake per minute (VO_2_max). Specifically, FM is negatively associated with VO_2_max, while FFM shows a positive association with it [17,18,19]. Moreover, BCM showed a higher positive association value with VO_2_peak than FFM [20,21].

Interestingly, VO_2_max reflects the maximal ability in terms of oxygen uptake, use, and transport [22]. It is a predictor of general health and fitness and is considered a key physiological measure both in the healthy [23] and the clinical population. However, no data are available on possible associations between body composition parameters and VO_2_max in patients with FSHD. A cardiopulmonary exercise test (CPET) on a treadmill or a cycle ergometer until exhaustion is typically used to estimate VO_2_max. However, the exercise intolerance phenomenon indicated by Vera et al. [15] suggests that execution of the test may be problematic for some patients. Moreover, for the 20% of patients who use a wheelchair [8], CPET would not be feasible. Therefore, given the well-known association of VO_2_max with health and functional domains in both the healthy population [24] and several clinical populations [25,26,27,28], the search for possible associations between maximal oxygen consumption and body composition parameters may help to identify additional, easily obtainable, anthropometric-based information aiming at the clinical stratification of patients, a pivotal factor to address the phenotypic variability observed in FSHD [29].

Among the most accurate techniques for evaluating body composition, dual-energy X-ray absorptiometry (DXA) is considered the reference method. However, since its application involves high costs [30], alternative methods such as bioelectrical impedance analysis (BIA) are commonly used for routine assessments [31].

The purpose of this study was to verify whether body composition parameters (FFM, FM, and BCM), obtained by a non-invasive and simple examination such as BIA, are associated with VO_2_max in FSHD patients.

## 2. Materials and Methods

### 2.1. Participants

The demographic and clinical characteristics of the participants are reported in Table 1. Inclusion criteria were as follows: age ≥ 9 years; clinical and genetic diagnosis of FSHD; registration in the Italian National Register for FSHD. Exclusion criteria were as follows: using a wheelchair at the time of selection; use of corticosteroids; severe cardiac and respiratory dysfunction; psychological or psychiatric disorders; major osteoarticular dysfunctions. Pediatric patients were included considering that, in subjects with early onset, the severity and speed of the disease are greater than in patients with classic onset [32]; therefore, it seems plausible to expect alterations in the considered parameters even at a young age. In total, 22 patients with FSHD were involved in the study. The sample was composed by 16 men and 6 women, with a mean age of 35.18 years (< >9–61 years). Of these, 17 patients belonged to the A clinical category and 5 to the B clinical category of the Comprehensive CCEF [12], as presenting facial and scapular girdle muscle weakness (category A) or muscle weakness concerning the scapular girdle or facial muscles (category B). All subjects provided written, informed consent to participate in this study (provided by parents or legal guardians for minor participants), which was conducted according to the Declaration of Helsinki (1975). This study was approved by the Lombardy Territorial Ethics Committee 6, protocol number 0006176/24 on 31 January 2024. The study was carried out at the CRIAMS-Sport Medicine Centre of Voghera (University of Pavia, Voghera, Italy).

### 2.2. VO_2_max Assessment

All participants performed a maximal cardiopulmonary exercise test (CPET) on a cycle ergometer (E 100, Cosmed, Rome, Italy) under electrocardiographic guidance, supervised by a medical doctor (GD), to check for cardiac events. A previous clinical incremental test to evaluate patients’ cardiac function allowed familiarization with such an experimental procedure. During the tests, pulmonary gas exchange was measured breath-by-breath using a face mask (V2 Mask TM, Hans Rudolph Inc., Shawnee, KS, USA) connected to a gas analyzer (Quark PFT, Cosmed, Italy). The test was performed with the step incremental technique (15 Watts (W) every 30 s, with a previous baseline cycling of 3.5 min at 25 W). The test was considered maximal when it met three criteria, as follows: respiratory exchange ratio (RER) > 1.1, ratio of perceived exertion (RPE) ≥ 8, and VO_2_ at plateau for at least 30 s. Notably, RER was derived from raw values, while VO_2_max was calculated as the average of the 30 s following achievement of RER = 1.1 and RPE ≥ 8. For each patient the test was performed at 11 a.m. in a room with a constant temperature of 23 °C.

### 2.3. Body Composition and Anthropometric Assessment

The BIA measurement technique was used to investigate body composition parameters. For this purpose, a single frequency impedancemeter was used (50 kHz) (BIA 101, Akern/RJL srl, Florence, Italy). For all the participants, the test was performed at 8.00 a.m.; to avoid disturbances in fluid distribution, subjects were instructed to abstain from food and drink for ≤2 h before the procedure. During the test, subjects were asked to lie supine for ten minutes, with limbs extended and abducted. After cleansing the skin with isotropic alcohol, four adhesive electrodes with low intrinsic impedance (Biatrodes Akern Srl, Florence, Italy) were placed on the back of the hands and four more electrodes on the neck of the corresponding feet. Before positioning, the skin, shaved if necessary, was gently abraded and cleaned with 75% alcohol to reduce electrical impedance. The parameters taken into account were weight (kg), BMI (kg/m^2^), FFM (kg), FM (kg), and BCM (kg). To account for body size, BIA parameters were also considered as indexed values (kg/m^2^). The estimates were obtained with Bodygram PRO v.3.0 software. To estimate FFM in children under 10 years old, the software uses the following equation: FFM = total body water (TBW)/0.755 [33], in which TBW is estimated using Kushner’s equation [34]. For the age range of 10 to 16 years, the software employs proprietary clinically validated equations for FFM [35]. In adults and geriatric populations, the equation for estimating FFM is from Sun et al., 2003 [36]. Regarding BCM, the software implements the equation BCM = 0.29 × FFM × Ln × Pha, which was clinically validated in [37,38]. Finally, FM was estimated by subtracting FFM values from body weights.

### 2.4. Statistical Analysis

Categorical variables were described through frequency distributions, and continuous variables were presented using mean and standard deviation. The relations between continuous variables were investigated using Spearman correlation coefficients (SCCs) to account for the small sample size. Statistical significance thresholds were set at 5%, 1%, and 0.1%. We conducted three sensitivity analyses by calculating the correlations for adult patients only, male patients only, and patients with type A FSHD only (the size of the subsamples of minors, females, and type B FSHD patients were too low, at *n* = 5, *n* = 6, and *n* = 5, respectively). All statistical analyses were carried out with Stata/IC v16.0 (StataCorp, College Station, TX, USA).

## 3. Results

### 3.1. Participants

Demographic and clinical characteristics are reported in Table 1. Patients had a mean FFM of 58.66 kg (±20.91), while the mean FM was 15.83 kg (±6.80), and the mean BCM was 24.45 kg (±8.33). The mean weight and height were 74.49 kg (±17.65) and 169.01 cm (±11.57), respectively, while the mean BMI was 22.85 kg/m^2^ (±4.25). Regarding indexed values, patients had a mean fat free mass index (FFMI) of 20.13 kg/m^2^ (±6), while the mean fat mass index (FMI) was 5.45 kg/m^2^ (±2.09), and the mean body cell mass index (BCMI) was 8.76 kg/m^2^ (±2.31).

The following mean values were obtained from the CPET: VO_2_max, 30.99 mL/min/kg (±9.87); oxygen consumption at anaerobic threshold (VO_2_AT), 20.71 mL/min/kg (±7.12); maximal breathing frequency (BF), 35.60 breath/min; maximal ventilation (MV) 66.79 L/min (±25.83); maximal tidal volume (TV) 1.97 L (±0.63); maximal heart rate (MHR) 163 beats per minute (BPM) (±13.79)(Table 1).

### 3.2. Correlation Analysis between Body Composition, Anthropometric Parameters, and VO_2_max

Correlations between VO_2_max and the other variables considered are summarized in Table 2. No significant correlations were observed between VO_2_max and FFM (SCC: 0.027), BCM (SCC: 0.075), FFMI (SCC: 0.02), BCMI (SCC: 0.067). 

**Figure 1:**
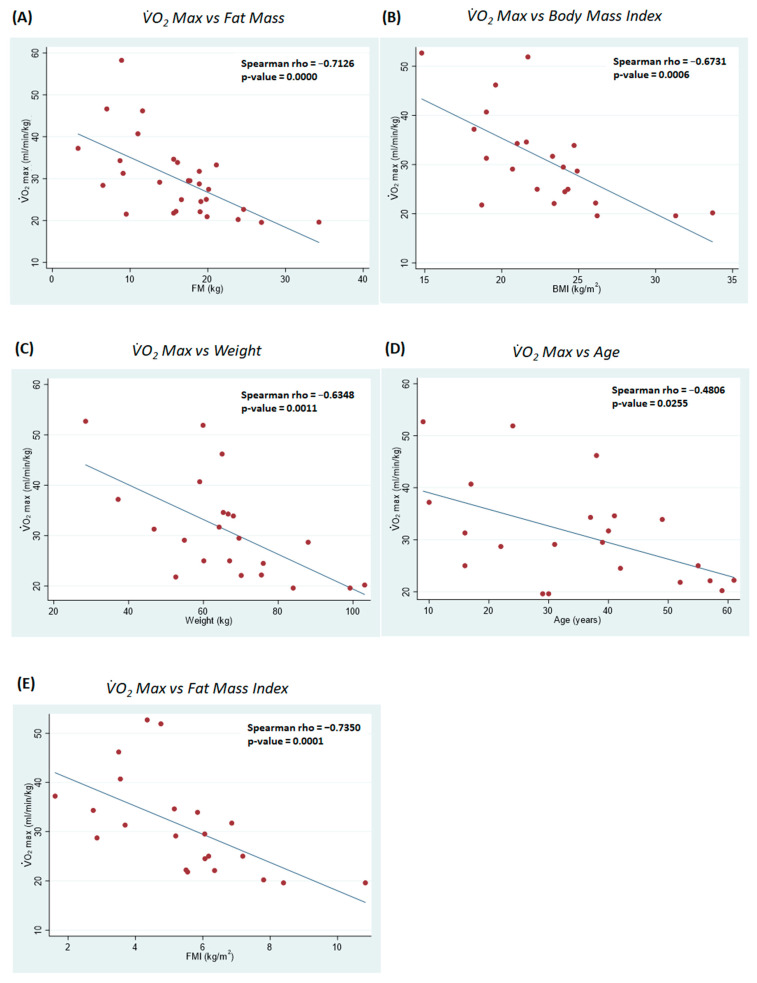
Scatterplots, relating to all participants, of the statistically significant correlations between VO_2_max and fat mass (**A**), body mass index (**B**), weight (**C**), age (**D**), fat mass index (**E**).

Instead, the analyses highlighted five statistically significant relations between VO_2_max and age (SCC: −0.48), weight (SCC: −0.635), FM (SCC: −0.712), FMI (SCC: −0.735), and BMI (SCC: −0.673); all these relations were negative, meaning that higher values of age, weight, FM, and BMI were related to lower VO_2_max levels. The graphic details of these relations are exposed in the scatter plots reported in Figure 1.

Considering adult patients only (*n* = 17), the significance of the SCC was confirmed for weight (SCC: −0.603), FM (SCC: −0.714), FMI (SCC: −0.719), and BMI (SCC: −0.595), but not for age (SCC: −0.269). Similar results were obtained for the subpopulation of males (*n* = 16; weight SCC: −0.904; FM SCC: −0.776; FMI SCC: 0.707; BMI SCC: −0.908) and type A FSHD subjects (*n* = 17; weight SCC: −0.712; FM SCC:−0.805; FMI SCC: 0.738; BMI SCC: −0.765).

## 4. Discussion

As far as we know, this is the first study analyzing the relationship between body composition parameters measured by BIA and VO_2_max values in patients with FSHD. The obtained results showed a negative association between VO_2_max and age, FM, body weight, and BMI; importantly, no associations were found between maximal aerobic fitness and FFM and BCM. The associations with the BIA parameters remained the same even when the values were indexed (Table 2).

In healthy subjects, a gradual decrease in VO_2_max has been reported with age [39,40]. Such a decrease is mostly linked to age-related change in body composition, which become worse when associated with a progressive increase in FM and a decrease in FFM during the aging process [41]. Our results seem in line with healthy subjects’ trends; however, the weak, although significant, association value indicates the need to test a larger sample of patients to confirm or deny this association. The weaker association between VO_2_max and age compared to body composition parameters could reflect the wide clinical variability of the disease [29], in which functional outcomes are not necessarily associated with age [42], while they often are associated with body composition [13,14,15]. In this light, our results seem to be coherent with the literature data. The association between FM and VO_2_max was found to be the strongest (see Figure 1); this seems to be consistent with some previous findings of a negative association between FM and VO_2_max in different populations of subjects [43,44,45], including a small group of patients with FSHD [15]. Considering the sample, it seems reasonable to interpret the BMI and weight associations as confirmatory data of the FM association, as in these patients an increase in weight, and therefore an increase in BMI, is plausibly attributable to an increase in FM [5,13,46].

The associations between VO_2_max and weight, BMI, and FM were also present considering adult patients only, male patients only, and category A patients only. Instead, age association lost significance in all subgroups. Since this association is the weakest of all those observed, the increase in variability caused by the reduction in sample size could be the reason for the loss of significance.

It remains unclear why a positive association between aerobic fitness and FFM and BCM was not found in our sample population, whereas several works reported it in healthy subjects [20,21,47,48].

The lack of such an association in our FSHD sample may be due to the disease-driven low amount of residual FFM and/or, possibly, to the associated shift in skeletal muscle composition. Indeed, in an interesting work by Celegato et al. [49], a fast-glycolytic to slow-oxidative muscle phenotype transformation, which is linked to a concomitant deficit of fundamental proteins involved in the response to oxidative stress, is reported; these data could suggest that the residual muscle mass in this patient cohort becomes progressively metabolically dysfunctional, which may not be associated with the overall aerobic fitness. Future studies should clarify if the level of training and/or of objectively measured physical activity can influence such an association in patients with FSHD.

Finally, in apparent contrast with our findings, a positive association with FFM was found in patients with FSHD by Vera et al. [15]. In their study, whose primary aim was to evaluate exercise intolerance in FSHD, an association of absolute and percent leg lean mass vs. VO_2_ peak was detected. This discrepancy may be due to the different methodologies used to estimate body composition (whole-body BIA vs. segmental dual-energy X-ray absorptiometry), to differences in the sample dimensions (22 vs. 11 subjects), or in clinical characterization of the patients. Furthermore, in our experimental conditions, body composition was associated to VO_2_max, while in the study of Vera et al. [15] it was associated to VO_2_peak.

Overall, our results indicate FM as the body composition parameter with the highest associative value with VO_2_max in patients with FSHD. This may help clinicians to better stratify patients. In fact, the negative association between FM and a fundamental functional indicator as VO_2_max seems in line with an interesting 14-year follow-up study, including 7142 adult subjects, which showed that adiposity is a predictive factor for the development of physical disability [50]. Our findings suggest that, in patients with FSHD, a high FM value could indicate a low level of physical efficiency and should be promptly addressed with ad hoc investigations and interventions, including tailored nutritional and/or physical exercise plans. Furthermore, even in patients who do not have high FM values, the inclusion in the clinical routine of strategies aimed at controlling body composition could be useful to maintain an acceptable level of physical efficiency long-term or to reduce the burden of deconditioning caused by diseases.

### Limitations and Future Directions

This study suffers from some limitations. First, bioimpedance measurements are based on predictive models that must be tailored to a particular cohort [51]. Although previously used in other muscular dystrophies [52,53,54,55,56], population-specific models for patients with FSHD have not been developed, and this may have, to some extent, biased the results. No control group was included in the present work; in future studies it should be present to compare VO_2_max and body composition values between patients and healthy subjects. Additionally, future studies on a larger sample will be needed to confirm or disprove the results obtained in the present work. Specifically, the sample size does not allow to support a multivariable regression analysis to evaluate the influence of possible confounders such as age, gender, or weight on the identified associations. Furthermore, only patients belonging to clinical subcategories A and B were included here, and the low number of B patients did not allow us to evaluate possible differences between these two clinical categories. To determine if variations in the clinical presentation of the disease are associated with variations of proposed associations, it is necessary to include all subcategories in future studies. Particularly, it will be of interest to test if, in patients with type C, a category composed of asymptomatic subjects, the association between FFM and VO_2_max is present, as in healthy subjects, or not; a possible absence could lead to research of metabolic alterations not yet detected in FSHD. Furthermore, the impact of the level of training and/or of objectively measured physical activity on the association between VO_2_max and body composition should be evaluated as well.

## 5. Conclusions

Our results indicate that VO_2_max is negatively associated with age, FM, BMI, and weight in patients with FSHD. The obtained data seem to reflect the resulting changes in body composition. Since FM is the body composition parameter that shows the highest association value with VO_2_max, it could be taken into account in the clinical stratification of patients with FSHD.

## Figures and Tables

**Table 1:** Descriptive statistics of the sample.

Variables		*n*	Mean	Standard Deviation	Percentual Values (%)
Socio-demographic variables					
Gender	Female	6	-	-	27.28
	Male	16	-	-	72.72
Age (y)		-	35.18	16.14	
FSHD variables					
FSHD category	A	17	-	-	77.27
	B	5	-	-	22.73
Anthropometric variables					
Weight (kg)		-	74.49	17.65	-
Height (cm)		-	169.01	11.57	-
Body Mass Index		-	22.85	4.25	-
Body composition variables					
Fat Free Mass (kg; % of BW)		-	58.66	20.91	78.74
Fat Free Mass Index (kg/m^2^)			20.13	6.00	-
Fat Mass (kg; % of BW)		-	15.83	6.80	21,26
Fat Mass Index (kg/m^2^)			5.45	2.09	-
Body Cell Mass (kg; % of BW)		-	25.45	8.33	34.16
Body Cell Mass Index (kg/m^2^)			8.76	2.31	-
Cardiopulmonary exercise test variables					
VO_2_max (mL/min/kg)		-	30.99	9.87	-
VO_2_AT (mL/min/kg)		-	20.71	7.12	-
Ventilation Max (L/min)		-	69.82	25.82	-
Breathing Frequency Max (b/min)		-	35.60	8.00	
Tidal Volume Max (L)		-	1.96	0.63	-
Heart Rate Max (beat/min)		-	163	13.79	-

FSHD, facioscapulohumeral muscular dystrophy; y, years; kg, kilograms; max, maximal; cm, centimeters; mL, milliliters; min, minute; b, breath; BW, body weight; VO_2_AT, oxygen consumption at anaerobic threshold; L, liter; m, meter.

**Table 2:** Spearman correlation coefficients between VO_2_max and the other continuous variables.

Variables	VO_2_max (mL/min/kg)			
	Total Sample (*n* = 22)	Adults (*n* = 17)	Males (*n* = 16)	Type A FSHD (*n* = 17)
Sociodemographic variables				
Age (y)	−0.48 *	−0.26	−0.46	−0.32
Anthropometric variables				
Weight (kg)	−0.63 **	−0.60 *	−0.90 ***	−0.71 **
Height (cm)	−0.31	−0.21	−0.43	−0.39
Body Mass Index	−0.67 ***	−0.59 *	−0.90 ***	−0.76 ***
Body composition variables				
Fat Free Mass (kg)	0.02	0.21	−0.15	−0.18
Fat Free Mass Index (kg/m^2^)	0.02	0.23	−0.10	−0.19
Fat Mass (kg)	−0.71 ***	−0.71 **	−0.77 ***	−0.80 ***
Fat Mass Index (kg/m^2^)	−0.73 ***	−0.71 **	−0.70 **	−0.73 ***
Body Cell Mass (kg)	0.07	0.05	−0.40	−0.15
Body Cell Mass index (kg/m^2^)	0.06	0.10	−0.34	−0.02

* *p* < 0.05, ** *p* < 0.01, *** *p* < 0.001; mL, milliliters; min, minute; kg, kilograms; m, meter.

## Data Availability

The data presented in this study are available on request from the corresponding author due to privacy and ethical reasons.
